# OptiScreen – ein Schulungskonzept für Pflegekräfte zur Durchführung des psychosozialen Distress-Screenings

**DOI:** 10.1007/s00761-023-01343-8

**Published:** 2023-04-28

**Authors:** L. Dreismann, M. Wenzel, V. Ginger, T. Zimmermann

**Affiliations:** Klinik für Psychosomatik und Psychotherapie, Medizinische Hochschule Hannover, Carl-Neuberg-Str. 1, 30625 Hannover, Deutschland

**Keywords:** Versorgung, Psychische Belastung, Training, Psychoonkologie, Kommunikationsschulung, Psychological distress, Cancer care, Training, Psycho-oncology, Communication training

## Abstract

**Hintergrund:**

Eine angemessene, bedarfsgerechte psychoonkologische Versorgung reduziert Depressivität und Ängste von Krebserkrankten sowie ihren Angehörigen und erhöht die Lebensqualität. Psychisch belastete Krebserkrankte werden jedoch nicht flächendeckend identifiziert, um ihnen psychoonkologische Unterstützung anzubieten. Screeningfragebögen haben sich zur Identifikation bewährt, allerdings bestehen bei der Anwendung im klinischen Alltag Hürden. Pflegekräfte haben durch ihren kontinuierlichen Kontakt zu Patient_innen, die vielfältigen klinischen Eindrücke und ihre Verbindung zu anderen Berufsgruppen eine Schlüsselrolle.

**Ziele:**

Die *OptiScreen*-Schulung soll Pflegekräfte in der Onkologie zur Durchführung des Distress-Screenings befähigen, entsprechendes Expert_innenwissen vermitteln und Hürden sowie Unsicherheiten im Screeningprozess abbauen, um somit belastete Erkrankte zielgerichtet identifizieren und einer psychoonkologischen Versorgung zuführen zu können.

**Das Training:**

Die *OptiScreen*-Schulung gliedert sich in drei Module à 1,5–2 h zu den Themen psychische Störungen bei Krebs, psychoonkologische Versorgung, psychische Belastung, Distress-Screening, Kommunikation im Screeningprozess und Psychohygiene (vermittelt durch Vorträge, Videos, Rollenspiele, Übungen).

**Ergebnisse und Diskussion:**

Erste praktische Erfahrungen weisen auf eine erfolgreiche Umsetzung des Schulungskonzepts hin. Weitere Ziele sind es, den Wissenszuwachs und die zunehmende Sicherheit der Pflegekräfte im Screeningprozess zu stärken sowie die Effekte langfristig aufrechtzuerhalten. Zusätzlich soll die Schulung in verschiedenen Settings etabliert und die Auswirkungen der Schulung in Bezug auf die Informiertheit und Zufriedenheit der Patient_innen mit dem Screeningprozess untersucht werden.

## Abstract (en)

**Background:**

Adequate, needs-based psycho-oncological care reduces depression and anxiety in cancer patients and their relatives, and increases quality of life. However, cancer patients experiencing psychosocial distress are not generally systematically identified in order to offer them psycho-oncological support. Screening questionnaires have proven useful for identification, but there are barriers to their use in clinical practice. Nurses play a key role due to their continuous contact with patients, diverse clinical experience, and interactions with various professional groups.

**Objectives:**

OptiScreen training is intended to enable nurses in oncology to perform distress screening, to impart appropriate expert knowledge, and to reduce hurdles and uncertainties in the screening process in order to identify distressed patients in a targeted manner and to provide them with psycho-oncological care.

**Training:**

OptiScreen training is divided into three modules of 1.5–2 h each on the areas of mental disorders in cancer, psycho-oncological care, psychological distress, distress screening, communication regarding the screening process and self-care (conveyed through lectures, videos, role playing, exercises).

**Results and discussion:**

Preliminary practical experience indicates successful implementation of the training concept. Further goals are to increase the knowledge and confidence of the nurses in the screening process and to maintain the effects in the long term. In addition, training will be established in different settings and the effects of the training on the patients’ awareness and satisfaction with the screening process will be assessed.

## Hintergrund

Das psychoonkologische (psychoonkolog.) Screening mittels validierter Screeningfragebögen ist in der Praxis gut erprobt und kann psychisch belastete Patient_innen (Pat.) erfolgreich identifizieren [1]. Ca. 50–70 % aller Krebserkrankten sind psychisch belastet [2, 3] und ca. ein Drittel weist im Laufe der Erkrankung eine psychische Störung auf [4]. Die daraus resultierende psychoonkolog. Behandlungsnotwendigkeit ist eindeutig. Trotz etablierter Screeningverfahren und psychoonkolog. Versorgungsstrukturen wird ein Teil der belasteten Pat. nicht identifiziert [5] oder adäquater Versorgung zugeführt [6, 7]. Die Gründe dafür sind vielfältig und liegen sowohl auf Pat.-Seite (Angst vor Stigmatisierung, Anspruch, sich selbst helfen zu wollen, fehlendes Wissen über Angebote etc. [8]) als auch auf Behandlungsseite (Zeitdruck, Wissensdefizite, geringer Stellenwert der Psychoonkologie etc. [9, 10]) oder sind struktureller Art (kurze Liegezeiten, keine ortsnahen Unterstützungsangebote, unklare Verantwortung etc. [9]). Deshalb fordern die S3-Leitlinie Psychoonkologie [11] wie auch die aktuelle Best-Practice-Empfehlung [12] sowie internationale Leitlinien (z. B. NCCN [13]) den Einsatz von Screeninginstrumenten inklusive Schritt-für-Schritt-Anleitung. Darüber hinaus ist das psychoonkolog. Distress-Screening seit 2022/2023 eine Kennzahl im Zertifizierungsprozess der Deutschen Krebsgesellschaft [14].

Im klinischen Alltag existieren dagegen heterogene Umsetzungen, Verantwortungsdiffusionen sowie Lücken in der Weiterleitung des Screeningergebnisses bzw. in der Einleitung der psychoonkolog. Unterstützung [15]. Diese Barrieren könnten jedoch reduziert werden. So ist die Inanspruchnahme für psychoonkolog. Angebote deutlich erhöht, wenn das Screening mit einem persönlichen Gespräch oder einer Empfehlung verknüpft wird [16]. Nach der Best-Practice-Empfehlung stellt der erste Schritt, die Identifikation psychisch belasteter Patient_innen mithilfe eines Screenings, keine Aufgabe der Psychoonkologie dar und obliegt der jeweiligen Klinik bzw. Station, die eine für das Screening verantwortliche Person benennen soll [10]. Der nächste Schritt, bei als belastet identifizierten Patient_innen eine weitere psychologische Diagnostik und entsprechende weitere Behandlung einzuleiten, ist Fachkompetenz und Aufgabe der Psychoonkologie. Häufig ist die Verantwortlichkeit für die Durchführung des Screenings nicht klar geregelt und wird von Pflegekräften, dem Aufnahme- oder Casemanagement übernommen [17], jedoch fehlen diesen ohne Schulung Informationen über aktuelle psychoonkolog. Unterstützungsangebote [18]. Zudem finden sich Unsicherheiten beim Behandlungsteam, psychische Belastungen anzusprechen, aus Sorge, nicht adäquat darauf reagieren zu können [19]. Abhilfe kann Weiterbildung sowie ein verbesserter interdisziplinärer Austausch schaffen, wobei insbesondere die Pflege in ihrer Funktion gestärkt werden sollte. Die Pflege in der Onkologie hat eine bedeutende Schlüsselrolle inne. Diese zeigt sich sowohl in der kontinuierlichen Begleitung sowie in vielfältigen klinischen Eindrücken von den Pat. als auch durch die Zusammenarbeit mit verschiedenen Berufsgruppen. Studien zeigen zudem, dass Pflegekräfte nach Kommunikationsschulungen psychisch belastete Pat. besser identifizieren konnten [20], sich in Interaktionen selbstsicherer fühlten [21] und ihr Wissen bzgl. psychischer Belastungen bei Krebserkrankungen erweitert wurde [22].

Internationale Distress-Management-Programme fordern, dass onkologisches Personal entsprechend geschult werden sollte, um die Qualität der Screeningdurchführung zu erhöhen und Hürden in der Implementierung abzubauen [13, 23]. Bisher gibt es in Deutschland jedoch keine Schulung für Pflegekräfte speziell zum psychoonkolog. Screening. Die durch die Deutsche Krebshilfe geförderte Multicenterstudie „OptiScreen“ („*Optimierte psychoonkologische Versorgung durch einen interdisziplinären Versorgungsalgorithmus – vom Screening zur Intervention*“; [24]) will diese Lücke schließen. „OptiScreen“ hat das Ziel, die psychoonkolog. Versorgung von Pat. zu verbessern, indem der Screeningprozess durch eine Schulung (*OptiScreen*-Schulung) und Einbindung der onkologischen Pflege unterstützt wird. Die entwickelte *OptiScreen*-Schulung soll Barrieren sowohl auf Seite des Behandlungsteams als auch auf Pat.-Seite abbauen, indem der Kommunikationsprozess rund um das Screening optimiert wird. Das Ziel des Schulungskonzepts liegt darin, der Pflege Wissen bzgl. des Screeningprozesses und psychoonkolog. Unterstützungsangebote zu vermitteln sowie Sicherheit in der Kommunikation innerhalb dieses Prozesses durch Training zu erreichen (z. B. Screeningfragebogen erklären oder zum Ausfüllen motivieren). Die zielgenaue Identifikation von belasteten Pat. sowie die bedarfsgerechte psychoonkolog. Versorgung sollen durch eine Kombination aus Screening und persönlichem Gespräch ermöglicht werden. Dabei übernimmt die Pflege nicht die psychoonkolog. Diagnostik, sondern kann in ihrer Schlüsselrolle als enge Begleitung der Patient_innen das Screening und Unterstützungsangebote vor Ort erklären und bei Belastung auch eine Empfehlung an den Patienten/die Patientin aussprechen oder zur Inanspruchnahme motivieren. Durch die *OptiScreen*-Schulung der Pflege wird die interdisziplinäre Zusammenarbeit gefördert und ein „Best-Practice-Modell“ entwickelt.

## Die OptiScreen-Schulung

### Konzeption

**Figure 1:**
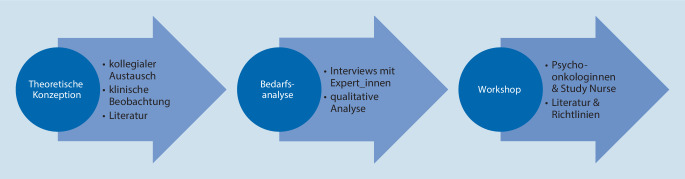

Die *OptiScreen*-Schulung wurde in aufeinander aufbauenden Schritten konzipiert (siehe Abb. 1). Nach einer Zusammenschau aus klinischen Beobachtungen, kollegialem Austausch, Literaturrecherche und in Abgrenzung zu bereits vorhandenen Kommunikationsschulungen erfolgte eine Bedarfsanalyse mittels Interviews mit Expert_innen aus der onkologischen Pflege [25]. Die Analyse des Schulungsbedarfs, inhaltlicher und didaktischer Hinweise, aktuell bestehender Hürden im Screeningprozess und der Einstellung der Pflege zu einer möglichen Schulung [18] schaffte die Grundlage für die Ausarbeitung des Schulungskonzepts. Im anschließenden Workshop mit Psychoonkologinnen wurden die einzelnen Module anhand von Literatur aufbereitet und als Schulungsmaterialien zusammengestellt. Der in der Schulung vermittelte Screeningprozess, orientiert sich an den internationalen Richtlinien sowie deutschlandweiten Best-Practice-Empfehlungen [12] und ist angepasst an die Rolle der Pflegekräfte in den einzelnen Durchführungsschritten (NCCN Distress Management Guidelines [13]). Eine Study Nurse überprüfte Verständlichkeit und praktische Relevanz aus Sicht der Pflege.

### Inhalte

**Figure 2:**
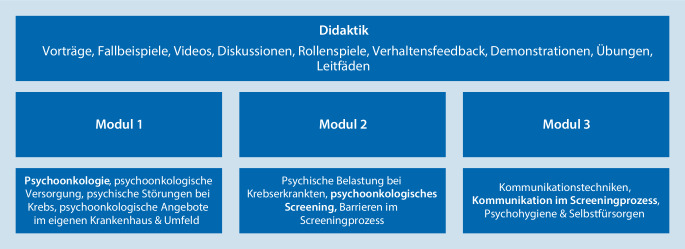

Die *OptiScreen*-Schulung gliedert sich in drei Module (siehe Abb. 2). Die Durchführung eines Moduls dauert 1,5–2 h und kann als Einzelschulung an drei Terminen oder als Schulungstag (mind. 6 h) durchgeführt werden. Ziel der Schulung ist es, Wissen bzgl. Psychoonkologie und häufiger psychischer Belastungen zu erlangen sowie die Anwendung und Auswertung des Screeningfragebogens der eigenen Klinik zu lernen, mithilfe von Kommunikationsfertigkeiten die Identifikation psychisch belasteter Erkrankter zu optimieren und sie durch gezielte Weitervermittlung an psychoonkolog. Angebote vor Ort einer bedarfsgerechten Versorgung zuzuführen.

### Didaktik

Die einzelnen Schulungsmodule sind didaktisch abwechslungsreich und mit Bezug auf die Bedürfnisse sowie erfahrungs- und teilnehmendenorientiert umgesetzt [25]. Dabei folgt die Methodik den Best-Practice-Empfehlungen zu Kommunikationsschulungen in der Onkologie [26]. Insbesondere die aktive Beteiligung der Teilnehmenden (Tn) an der Schulung wird zu Beginn fokussiert und es wird immer wieder dazu ermuntert. Als Struktur dient eine Themenübersicht zu Beginn jedes Moduls mit einer Agenda für die Tn sowie Zwischenfazit. Die theoretischen Inhalte werden anhand von präsentationsgestützten Vorträgen mit Fallbeispielen und Abbildungen (siehe Tab. 1) vermittelt. Eigene Beiträge und Erfahrungen der Tn können jederzeit eingebracht und zu verschiedenen Themen/Fragen innerhalb von Diskussionen im Plenum vertieft werden. Die einzelnen Schritte der Screeningdurchführung werden mittels Demonstrationen, Videobeispielen, Leitfäden und Rollenspielen (mit Verhaltensfeedback der Trainerinnen) aktiv eingeübt. Eine kleine Gruppengröße ermöglicht individuelle Rückmeldungen während der Schulung. Ein wichtiger didaktischer Baustein ist die kontinuierliche Hervorhebung der Transfermöglichkeit des Gelernten in den klinischen Alltag der Tn. Ergänzend werden Übungen zur Selbstfürsorge angeleitet. Nach jedem Modul werden Take-Home-Messages und Fragen gemeinsam festgehalten.

**Table 1:** 

Material	Inhalt/Ziel	Modus (gedr. = ausgedruckt, digi. = digital versandt)
*Schulungsordner*	Sammlung der Materialien pro Modul	Alle Tn erhalten Ordner
– Zeitplan	Themen & Zeiten für jedes Modul	Gedr.
– Schulungshandbuch	Ausführliche Beschreibung der Schulungsinhalte	Gedr. & digi.
– Präsentationsfolien	Folien aus der Schulung	Gedr. & digi.
– Screeningbogen	In der Klinik verwendete psychoonkolog. Screeningfragebögen	Gedr. & digi. inkl. Anleitung, wo auf den klinikinternen Laufwerken zu finden
– Zertifikat	Teilnahmezertifikat für die Gesamtteilnahme/einzelne Module der Schulung	Für die teilnehmende Station/den Arbeitsbereich gedr. & gerahmt & als personalisiertes Zertifikat für alle Tn gedr.
*Trainingsordner*	Anleitung & interne Hinweise zur Durchführung der Schulung	Trainer_innen erhalten digi. Ordner mit allen Vorlagen für die Schulung zur Anpassung
– Interner Zeitplan	Themen & Zeitplan mit genauer Folienanzahl & Dauer der Untereinheiten	Vorlage für drei Termine/einen ganzen Schulungstag
– Trainingshandbuch	Ausführliches Trainingshandbuch zu einzelnen Modulen mit Hinweisen zu Übungen, individuelle Anpassung an die eigene Klinik	PDF-Dokument
– Vorlage Präsentationsfolien	Präsentationsfolien der einzelnen Module zur eigenen Bearbeitung, Platzhalter für Individualisierung	PPT-Folien
– Vorlage Materialien	Vorlage für Zeitpläne, Anleitungen, Abbildungen, Evaluationen	Word-Dokument

## Durchführung

Die *OptiScreen*-Schulung wurde von Januar bis März 2021 an der Medizinischen Hochschule Hannover durchgeführt. Aufgrund der COVID-19-Pandemie wurde die Schulung in Kleingruppen von 4 bis 12 Tn realisiert und alle erhielten kleine Lunchpakete. Drei Trainerinnen führten an insgesamt 35 Terminen die *OptiScreen*-Schulung durch. Die Trainerinnen waren psychologische Psychotherapeutinnen und Psychoonkologinnen, was wichtig war, um auf praxisrelevante Fragen, z. B. zur psychoonkolog. Tätigkeit oder zu psychischen Störungen, eingehen zu können. Vor dem ersten Schulungstermin erfolgte die Präevaluation (*Opti-Eva* [26]) und nach dem letzten Modul die Postevaluation, mit dem Ziel der Komplementierung des hierarchischen Modells von Kirkpatrick zur Trainingsevaluation [27] und der Qualitätskontrolle der *OptiScreen*-Schulung.

### Teilnehmende (Tn)

An den drei Modulen nahmen insgesamt *N* = 72 Pflegekräfte (*n* = 62 weiblich, Alter *M* = 42,56, *SD* = 11,66, *Range* = 20–64) und somit die Grundgesamtheit (*n* = 65 Gesundheits- & Krankenpflege, *n* = 5 Casemanagement, *n* = 2 unbekannt) von drei viszeralonkologischen Stationen teil. Es gab keine Ausschlusskriterien. Die Tn absolvierten die Schulung innerhalb ihrer Arbeitszeit und konnten sich diese als Fortbildungspunkte anrechnen lassen. Fünf der Tn hatten zuvor bereits eine onkologische Fachweiterbildung und insgesamt vier Personen zuvor eine Fortbildung zur Psychoonkologie absolviert.

### Schulungsmaterial

Ein Schulungsordner enthält eine Ausarbeitung für jedes Modul (siehe Tab. 1).

## Diskussion

Die Pilotierung der OptiScreen-Schulung an der Medizinischen Hochschule Hannover wurde erfolgreich durchgeführt. Die Konzeption ist nach den Erfahrungen der Trainerinnen und aufgrund der positiven Beurteilung der Tn gut an den Schulungsbedarf der onkologischen Pflege angepasst. Langfristige (Neben‑)Effekte der Schulung z. B. auf die Teamatmosphäre oder Stressreduktion werden untersucht. Um die Effektivität analog zur Empfehlung zur Evaluation von Kommunikationsschulungen in der Onkologie auf verschiedenen Ebenen zu belegen, steht die Auswertung auf Pat.-Ebene aktuell noch aus [28]. Aus den klinischen Rückmeldungen des psychoonkolog. Teams zeigen sich positive Effekte bzgl. der Qualität der Konsilanfragen sowie Informiertheit der Pat. auf den Stationen, die an der Schulung teilgenommen haben. Zusätzlich bietet die kontinuierliche Feedbackschleife durch die Study Nurse eine aktive Auseinandersetzung mit dem Thema Screening. Dennoch bleibt zu beobachten, wie die langfristige Implementierung der Schulung und des Screenings gefördert werden kann. Ein möglicher Anreiz könnte sein, regelmäßige Schulungen zum Thema Screening für das Team in den Zertifizierungsrichtlinien zu verankern oder im internen Weiterbildungsprogramm zu etablieren.

Es bleibt zu beachten, dass die OptiScreen-Studie eine erste Pilotierung des Schulungskonzepts darstellt und in weiteren Kliniken sowie Settings und Fachbereichen validiert werden sollte. Hier könnten insbesondere der Personalnotstand und somit eine Freistellung während der Arbeitszeit für die 6‑stündige Schulung sowie volle Weiterbildungspläne Barrieren an anderen Klinikstandorten darstellen. Hier sollten der Wunsch und Bedarf aus der Pflege heraus nach dieser Schulung sowie der Mehrwert für die Pflege betont und die Terminierung in Rücksprache mit den Pflegenden geplant werden.

Nichtsdestotrotz bleiben einige strukturelle Hürden bestehen, die mittels Training alleine nicht zu bewältigen sind. Eine wichtige Verbesserungsmöglichkeit der Screeningimplementierung liegt in der verfügbaren Zeit für das Screening sowie Training des Personals [17]. Auch bei Implementierung elektronischer Screenings bleibt der Bedarf nach einer Schulung bestehen, da die Durchführung des Screenings nicht unbedingt zu einer Weiterleitung zu bedarfsgerechten Angeboten führt [29]. Es braucht in interdisziplinären Teams verantwortliche und geschulte Personen, die diesen Versorgungsalgorithmus aktiv betreiben, denn eine aktuelle Studie aus Deutschland zeigt, dass in den Zertifizierungsaudits sogar innerhalb der Kliniken heterogene Begründungen für mangelnde psychoonkolog. Betreuungsquoten in Abhängigkeit von verantwortlichen Personen gegeben werden [30]. Die persönliche Aufklärung über das Screening sowie die Empfehlung zu Unterstützungsangeboten bleibt weiterhin eine zentrale Aufgabe des onkologischen Teams und benötigt entsprechende Weiterqualifikation sowie Expertise, die die *OptiScreen*-Schulung bieten kann.

## Ausblick

Zur externen Validierung wird die *OptiScreen*-Schulung in anderen Kliniken durchgeführt und evaluiert. Die Ausweitung der Schulung auf andere Berufsgruppen (ärztliches Personal, Pflegeberatung) ist geplant.

## Fazit für die Praxis

Eine bedarfsgerechte psychoonkolog. Versorgung ist Teil einer umfassenden und modernen Onkologie. Psychisch belastete Krebserkrankte zielgerichtet identifizieren zu können und einer psychoonkolog. Versorgung zuzuführen, ist eine wichtige Aufgabe in der Onkologie. Die Anwendung von Screenings hat sich als zielsicher erwiesen, steht in der klinischen Praxis jedoch einer Reihe von Barrieren gegenüber. Die *OptiScreen*-Schulung für die Pflege in der Onkologie baut diese Barrieren ab, indem Wissen zu Psychoonkologie, psychischen Belastungen und Screening sowie Kommunikation erfolgreich vermittelt werden und sich somit die psychoonkolog. Versorgung verbessern kann.
